# Surveillance of Patients in the Waiting Area of the Department of Emergency Medicine

**DOI:** 10.1097/MD.0000000000002322

**Published:** 2015-12-28

**Authors:** Pia Hubner, Andreas Schober, Fritz Sterz, Peter Stratil, Christian Wallmueller, Christoph Testori, Daniel Grassmann, Nitaya Lebl, Iris Ohrenberger, Harald Herkner, Chirstoph Weiser

**Affiliations:** From the Department of Emergency Medicine, Medical University of Vienna, Austria.

## Abstract

Many patients visiting an emergency department are in reduced general condition of health and at risk of suffering further deterioration during their stay. We wanted to test the feasibility of a new monitoring system in a waiting area of an emergency department.

In an observational cross-sectional single-center study, patients with acute cardiac or pulmonary symptoms or in potentially life-threatening conditions were enrolled. Monitoring devices providing vital signs via short range radio (SRR) at certain time points and compliance evaluation forms were used.

Out of 230 patients, 4 wanted to terminate their participation prematurely. No data was lost due to technical difficulties. Over a median monitoring period of 178 (118–258) min per patient, 684 h of vital sign data were collected and used to assist managing those patients. Linear regression analysis between clinical symptom category groups of patients showed significant differences in the respiratory rate and noninvasive blood pressure courses. Feedback from patients and users via questionnaires showed overall very good acceptance and patients felt that they were given better care.

To assist medical staff of an emergency department waiting area to rapidly response to potentially life-threatening situations of its patients, a new monitoring system proved to be feasible and safe.

## INTRODUCTION

Many patients visiting an emergency department are in reduced general condition of health and at risk of suffering further deterioration during their stay at the emergency department. Those patients after initial screening of their complaint (triage) and defined as not seriously ill are usually asked to take place in the waiting area. They are not continuously monitored, and detection of acute health deterioration relies upon judgment of medical personnel alone, which, due to increasing patient numbers, can be challenging. Deterioration which is overlooked might lead to a life-threatening condition. Data strongly supports the notion that an in-hospital cardiac arrest is often a predictable event.^1^ Patients will frequently exhibit physiological signs of instability in the respiratory, cardiovascular, and/or neurological systems that can be recognized by routine patient monitoring and used to alert physicians.^2^ Unfortunately, because patients outside of high acuity areas of the hospital, such as the intensive care unit, are often not continuously monitored, these early indicators of an imminent serious adverse event are often missed. This can then lead to a serious patient deterioration that might have been recognized and treated earlier, had more vigilant physiologic monitoring been done. There are no studies reporting that a specific monitoring system has been tested at a waiting area of an emergency department, a setting where many patients are in the potential critical condition and often have to wait for several hours without being monitored.

The primary objective of the study was to learn if Philips IntelliVue Guardian Solution (IGS^1^) (Philips Medizin-Systeme Böblingen GmbH, Boeblingen, Germany), a less sophisticated telemetry monitoring system with focus on vital signs (blood oxygen saturation [SpO2], pulse, respiratory rate, and noninvasive oscillometric blood pressure measurements) while not having the patient directly connected to a conventional patient monitor can provide assistance when caring for such patients.

Emergency department waiting area patients most often have benign, self-limited conditions. Thus we expected the probability to catch a patient's deterioration into a serious condition of being very low within an affordable realistic time frame. It was also not our intention to present if the initial screening of emergency department patients (triage) is sufficient and makes such a system unnecessary. Therefore we decided to conduct as the initial step an observational study testing the feasibility and safety of using a new wireless vital sign monitoring system in the waiting area of an emergency department.

## MATERIALS AND METHODS

The study was conducted as an observational cross-sectional single-center study at the Department of Emergency Medicine at the Medical University of Vienna from July 2014 until November 2014.

The investigation complied with the Declaration of Helsinki's principles for physicians engaged in biomedical research involving human subjects and was approved by the appropriate ethics committee (EK #1552/2013, Ethikkommission, Medizinische Universität Wien); all subjects provided informed consent to participate. The monitoring devices tested together with the Philips IntelliVue Guardian Solution consisted of cableless measurement devices that provided blood oxygen saturation (SpO2), pulse, respiratory rate, and noninvasive oscillometric blood pressure measurements via short-range radio (SRR) technology to the IntelliVueCableless Hotspot while not having the patient directly connected to a conventional patient monitor (Philips IntelliVue Guardian Software, Philips IntelliVue MP5SC spot check monitor, Philips IntelliVue CL SpO2, Philips IntelliVue CLNBP, Philips IntelliVue CL respiration pod and Philips IntelliVue CL infrastructure).

The reasons to conduct as the first step only a feasibility and not comparative trial were: the limited resources with regard to patients obtainable and eager to participate in the project, the supplies available and the world wide first time acquiring of totally new knowledge with monitoring in the waiting area of an emergency department. Therefore, we only hypothesized if this monitoring system is easy to use and accepted by patients and users and that an ongoing continuous monitoring of patients vital signs is feasible and provides online information concerning their condition during their stay in the waiting area of an emergency department.

### Participants

Our emergency department takes care of ∼90,000 patients per year. Characteristics of patients showing up vary significantly. Upon arrival, patients routinely have their vital signs measured (blood pressure, SpO2, pulse rate, and body temperature) and are assessed regarding their current condition. For this initial assessment, the Emergency Severity Index (ESI),^3^ a 5-level emergency department triage algorithm, is used. It provides a clinically relevant stratification of patients into 5 groups from 1 (most urgent) to 5 (least urgent) on the basis of acuity and resource needs. If patients are stable and in overall good physical condition, they are asked to take place in the waiting area, where they will be seen by a physician as soon as possible. The decision to include patients was based on the ESI (eg groups 4 and 5), the inclusion criteria, consenting by the patients and on the available devices during this pilot trial.

### Inclusion Criteria, Phase 1

- Adult patients (18 years or older).
- Triaged as ESI 2 and 3.
- Conscious and able to give informed consent.
- At high risk of suffering from a cardiovascular deterioration, that is, patients with chest pain, blood pressure dysregulations, absolute arrhythmias, and/or heart failure New York Hearth Association classification 3 (NYHA), and/or.
- Have shortness of breath.

### Inclusion Criteria, Phase 2

After completion of 103 patients due to low enrolment rates, inclusion criteria were widened and gave the investigators more liberty to enroll also patients if they had the feeling that they might suffer from a potentially declining state of health risk.

Patients enrolled in the study were monitored as long as they stayed at the waiting area of the emergency department. Enrolled patients had to wear Philips monitoring devices (IntelliVue Cableless Measurement pods). The devices consisted of a cuff for blood pressure measurement, placed either on their upper arm or forearm, a finger photoplethysmography sensor for measurement of heart rate and SpO2, and a sensor attached to the left costal arch on the patient's chest for monitoring of respiratory rate. The pulse rate was not only recorded from photoplethysmography but also by blood pressure measurement and respiratory rate sensor consisting of an accelerometer. Vital signs were measured at set points in time, sent directly via cableless hotspot and local area network (LAN) infrastructure to the Philips IntelliVue Guardian Software, and analyzed there for possible deterioration. IntelliVue Guardian Software clients for observation were placed at the place for initial assessment (triage) of patients and in break room of the emergency department. Measurement of respiratory rate, pulse rate, and SpO2 initially took place every 15 min and blood pressure every 30 min. If necessary, time periods between measurements were adjusted according to the patient's condition.

At the end of data collection, when the patient was released from the hospital or admitted to a different department, patients and personnel handling the devices completed a questionnaire of 17 questions, which were formulated to evaluate the patients and users opinions and experiences.

### Statistical Analysis

Our aim was to assess the number of events per visit, per patient, and per hour waiting time in the outpatient unit. Continuous data are shown as mean and interquartile range (IQR), discrete data as counts and percentages. Data are presented with Microsoft Excel (2010 Microsoft Corporation, Mountain View, CA). No data were considered spurious in the analysis since all data were checked and cleaned before analysis. A subject screening and enrolment log was completed for all eligible or noneligible subjects. To test for differences in readouts we used linear regression models. The dependent variable was each readout, the covariable was clinical symptom category, modeled as an indicator variable with the most appropriate category as baseline. To allow for the panel structure of the dataset due to multiple measurements within each patient, and given a variable number of measurements we used a random effects linear model with patient id as the panel identifier. For data management and analysis we used MS Excel 2011 and Stata 14 for Mac (Stata Corp., College Station, TX). Generally we considered a 2-sided *P* value <0.05 statistically significant.

## RESULTS

From July 2014 until November 2014, a total of 230 patients were enrolled in the study. Of these 4 patients wanted to discontinue their participation prematurely and were, therefore, excluded from the study. Patients who did not wish to continue their participation explained they did not want to take part in a clinical study any longer or felt bothered by wearing the study devices. As there were no technical difficulties and we received usable data from all included patients, we concluded the sample size of 226 patients sufficient enough for data analysis; no data were missing.

In phase 1, 103 patients were enrolled. There were no dropouts in this group. Discharged after their visit to the ED were 87 (84%) patients, admitted to the hospital 16 (16%).

In phase 2, 127 patients were enrolled. Dropouts in this group were 4 (3%). Discharged after their visit to the ED were 91 (74%), admitted to hospital were 32 (26%) patients.

**FIGURE 1:**
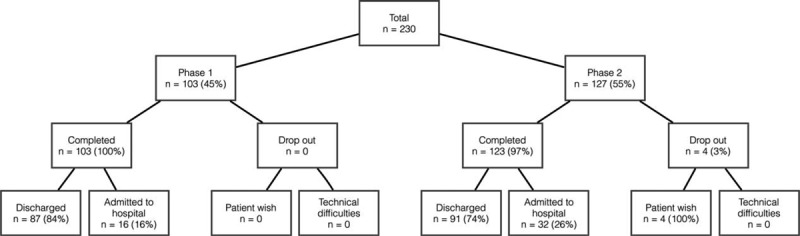
Flowchart—overview of participants.

Altogether (phase 1 and phase 2), discharged from hospital after their visit to the ED were 178 (79%) patients, admitted to hospital were 48 (21%) (Fig. 1).

Of 226 patients, men were 124 (55%) and the median age was 55 (43–71) years. The chief complaint on admission to the ED was chest pain in 125 (55%) patients, hypertension in 31 (14%), tachycardia and palpitations/arrhythmia in 28 (12%), dyspnea in 25 (11%), a situation after collapse in 14 (6%), and other symptoms in 3 (1%) patients (Table 1).

**TABLE 1:**
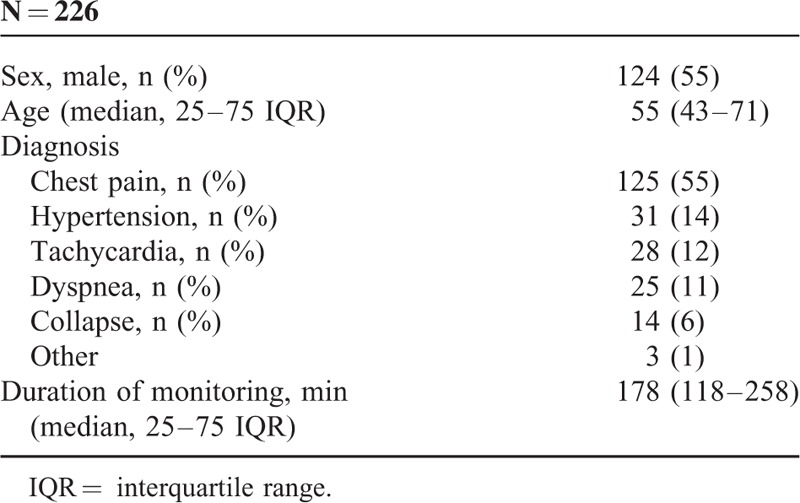
Epidemiology of Patients

### Monitoring

**FIGURE 2:**
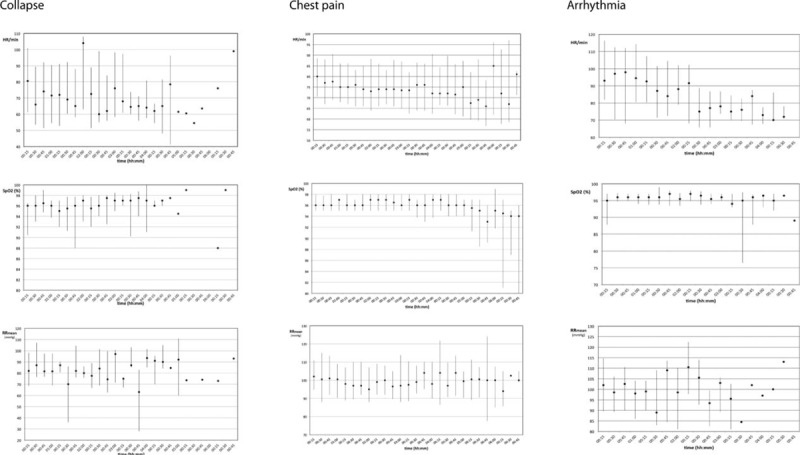
Pulse rate and SpO2 data from patients suffering collapse (n = 14), chest pain (n = 125), and arrhythmia (n = 28); data are presented as median and their interquartile range. SpO2 = blood oxygen saturation.

**FIGURE 3:**
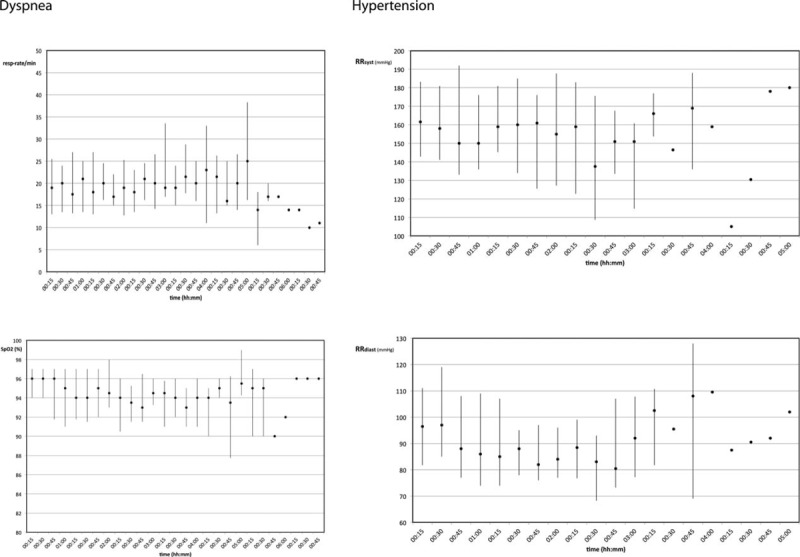
Respiratory rate and SpO2 data from patients with dyspnea on the left (n = 25); blood pressure data (systolic and diastolic; n = 31) from patients with hypertension on the right; data are presented as median and their interquartile range. SpO2 = blood oxygen saturation.

Altogether, 684 h of data were collected. Enrolled patients were continually monitored a median of178 (118–258) min. All monitored data consisted of pulse rate, SpO2, respiratory rate, and noninvasive oscillometric blood pressure measurements (Figs. 2 and 3). Pulse rate data were generated from pulse oximetry, blood pressure, and respiratory measurements with no differences between the techniques of measurement.

Blood oxygen saturation showed significant differences during the observational period for those patients having dyspnea as chief complaint compared only to those with chest pain (*P* = 0.022). Those patients having hypertension were significantly different in their blood pressure course to all other groups with chest pain (*P* < 0.001), palpitations (*P* < 0.001), dyspnea (*P* = 0.004), and collapse (*P* < 0.001). Similar all patients with dyspnea had significant different respiratory rates over time compared to all other groups of patients (*P* < 0.001). Interestingly heart rate over time showed only significant differences, if patients with palpitations were compared to those with chest pain (*P* < 0.001) and collapse (*P* = 0.024).

### Questionnaires

**FIGURE 4:**
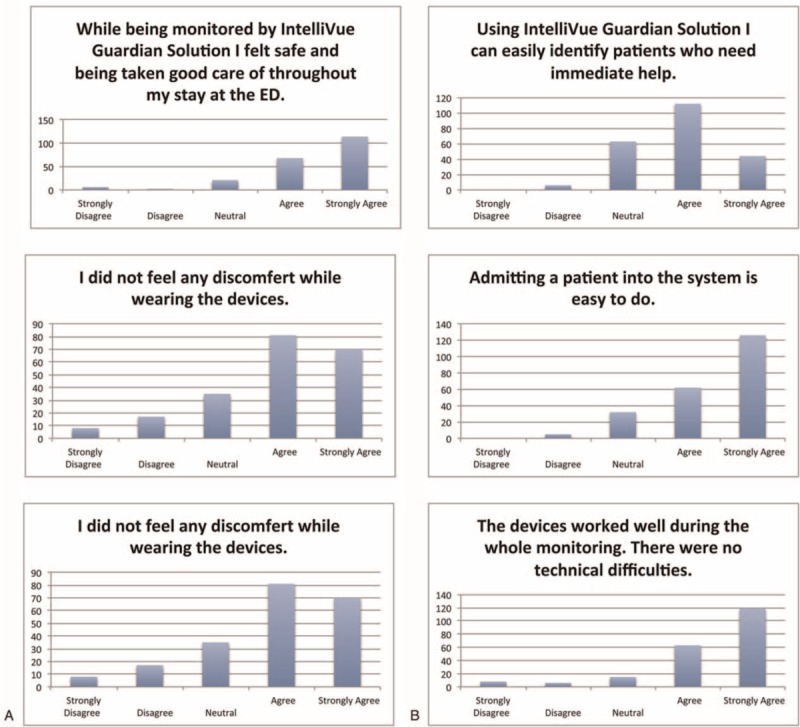
Questionnaire—A patients; B users (%).

Of all cases, 211 (93%) patients answered the questionnaire. When asked whether they felt safe and taken good care of while they were wearing the study devices during their stay at the ED, strong agreement to the question was found in182 (86%) patients, 21 (10%) had a neutral response, and 8 (4%) patients disagreed or strongly disagreed with this statement. No device-related discomfort was indicated in 151 (72%) cases, 35 patients (17%) gave a neutral response, and complaints about discomfort were found in 25 (12%) patients. Patients had the impression that the study devices worked well while wearing in182 (86%) cases, whereas 15 (7%) patients gave a neutral response, and disagreement to this question was found in 14 (7%) patients (Fig. 4a).

Users answered questionnaires in 225 (99%) cases. When asked if they could easily identify patients who needed immediate help via this monitoring technique, users agreed or strongly agreed in 156 (69%) cases, a neutral feeling was stated in 63 (28%) cases, and in 6 (3%) cases it was indicated that it was not easy to identify patients who need immediate help was the opinion during monitoring of 6 (3%) cases. Users stated the interpretation of vital signs is obvious and requires no assistance in 118 (52%) cases, neutral felt 75 (33 %), and problems with the interpretation of vital signs were reported in 32 (14%) cases. To admit patients to the monitoring system was easy in 188 (84%) cases, neutral in 32 (14%), and not simple in 5 (2%) patients (Fig. 4B).

## DISCUSSION

To assist medical staff of an emergency department waiting area in rapid response to life-threatening situations of its patients, a new monitoring system proved to be feasible and improved patient surveillance. Through collecting data of particular patients (n = 226) via the monitoring system, observational recordings and questionnaires for patients, nurses and doctors the investigation of the new observational system showed an exceptional satisfactory acceptance. Only 4 of the 230 enrolled patients dropped out due to their personal request during the course of monitoring. The study devices worked well in all cases; there were no dropouts due to technical difficulties, no issues concerning safety, no adverse events. A total of 684 h of monitoring data consisting of SpO2, pulse, respiratory rate, and noninvasive oscillometric blood pressure measurements were collected. During the observation of our 226 “Emergency Severity Index 2 & 3” patients there have been no urgent clinical patient management decisions necessary either based on clinical or monitored data. This might have been due to the careful selection of our patients based on the inclusion and exclusion criteria. Due to the feasibility/pilot character of our project, too short time and less patients have been studied to get an answer to the important question how many patients could be “rescued” due to the new monitoring strategy in the waiting area of our ED. But we assume that in our ED with a yearly visit of nearly 100,000 patients per year with an appropriate equipped waiting area of 15 devices 1 patient per year will be “rescued” and 1 patient per day will be “caught” before her/his condition will aggravate into a life-threatening condition. However, this will also depend on the human interface at the triage nursing classification and available observation care unit resources. So, as less observation care monitoring possibilities you have as more patients might benefit from such monitoring devices in the waiting area. Unfortunately we were also not able to present data on actual patient outcomes (eg how many had serious deterioration, how many potentially dangerous events were detected, etc). This would have been impossible within realistic affordable period of time.

The figures generated from the recorded monitoring data demonstrated changes in vital signs of patients during their visit at the emergency department (Figs. 2 and 3). These recordings otherwise would have been necessary during spot-check rounds of nurses closely monitoring the patients. Pulse rates recorded from separate sites—photoplethysmography sensor, blood pressure measurement, and respiratory rate sensor—showed that pulse rate values from SpO2 sensors and blood pressure are more or less coherent, whereas pulse rate values taken from respiratory rate sensors deviate, indicating pulse rate measurement from photoplethysmography and blood pressure measurement to be more precise than from accelerometry in the respiratory sensor.

Questionnaires indicated overall very good acceptance of the monitoring system by both groups, patients and users (Fig. 4), showing that Philips IntelliVue Guardian solution (IGS) and cableless measurement devices were well received in our setting. The majority of patients (86%) stated they felt save and taken good care of while wearing the study devices during their stay at the ED. The majority of users (69%) stated they could easily identify patients who needed immediate help.

Of all patients (phases 1 and 2), 178 patients (79%) were discharged from hospital after their visit to the waiting area of the emergency department, which shows that many of the patients do not need hospital admission. Patients are first examined at our triage, where their condition is evaluated by well-trained nurses. In most cases, they detect acutely sick patients and immediately triage them to a unit with continuous monitoring. As we only enrolled patients triaged to the waiting area, the number of hospital admissions of the enrolled patients was quite low (Fig. 1).

As patient vital signs are routinely only checked once— at admittance to our emergency department—deterioration of vital signs could easily be overlooked. An emergency department like ours is highly frequented by many acutely sick patients, who at arrival, have to be evaluated concerning their condition. During the triage evaluation of these patients, clinicians seldom have a complete medical history available and can often only judge patients’ medical threat within minutes upon their current state of health. In enrolled patients, vital signs were not only measured upon arrival of the patient at the outpatient department, but also at set points in time. Deterioration of vital signs of patients and acute treatment effects have been easily recognized by the personnel handling the devices. Therefore, the ongoing collection of vital signs after triaging patients has proved in the enrolled patients to be quite helpful for the medical management in our outpatient clinic. Thus the monitoring devices not only detected worsening conditions of patients, but also checked if a patient's therapy had the desired effect. In patients with hypertension it was possible to narrowly record their blood pressure after they had received antihypertensive therapy. Pulse rates of patients with arrhythmias or tachycardia who received frequency-regulating therapy could be checked at any time wirelessly. In Figure 2, displaying pulse rates of patients with arrhythmia or tachycardia, it is shown that initially high pulse rates declined during the patients stay. This was probably due to frequency regulating treatment patients received. Patients with ongoing atrial fibrillation, for example, would previously have been immediately admitted to our acute care unit to receive more invasive frequency-regulating therapy such as cardioversion. Now, continuously monitored with the new system, they were kept at the outpatient clinic, treated and given the chance to spontaneously convert to sinus rhythm without being placed on a monitor bed.

Studies have shown that an increased number of physiological abnormalities in critically ill emergency department patients is associated with increased mortality.^4–8^ Many patients who suffer in-hospital cardiac arrests present with abnormal physiological parameters before the event: tachypnoea (58%), tachycardia (54%), altered mental state (46%), arterial hypotension (46%), poor urine output (29%), pyrexia (13%), arterial hypertension (8%), and hypothermia (4%).^9^ In others it is indicated that the most frequent clinical deterioration seen before cardiac arrest is impaired respiratory or mental function, with the respiratory rate elevated well above normal in a majority of patients.^2^ Numerous studies have suggested the importance of measuring vital signs, particularly among patients at risk of adverse events.^2,5,6,10–11^ As patients visiting the emergency department for acute medical problems are often critically ill, continuous monitoring of these patients can prove to be highly important. However, even if patients’ vital signs are monitored, these changes have to be acknowledged by medical personnel. Many studies observed that avoidable arrests result from a failure to act on clinical information rather than a lack of information.^2,12^ This is a very important issue to discuss. A monitoring system like IntelliVue Guardian Solution(IGS) can only help detect deterioration in patients if personnel handling the devices and monitors quickly act upon changes in vital signs of patients. Hodgetts et al analyzed data from 118 sudden in-hospital cardiac arrests on adult patients admitted to hospital where resuscitation was attempted, and concluded that probably 73% of sudden cardiac arrests had likely been avoidable and that clinical signs of deterioration in the preceding 24 h had often not been acted upon.^1^ No cardiac arrest occurred in our outpatient clinic during our study period and was a very rare event in recent years, which makes it difficult to form assumptions based on our data.

Many studies have dealt with the introduction of medical emergency teams (MET) to treat deteriorating patients on general wards as soon and efficiently as possible.^13,14^ In some settings, a scoring tool such as the multiparameter Modified Early Warning Scoring tool (MEWS) was introduced as an additional trigger to activate the MET instead of single parameter triggers and has shown to perform well for prediction of cardiac arrest and death within 48 h.^15,16^ MEWS is a scoring system that we believe has yet to be adapted to be more of use in a setting like an outpatient department and in our opinion should be expanded to include telemetric ECG-monitoring. As patients’ symptoms and underlying diseases can vary significantly in this setting, we believe a scoring system would have to be adapted to different patient groups. For example, patients with tachycardia but otherwise no acute problems should have their pulse rate especially monitored. Monitoring and scoring of patients with respiratory problems should focus on SpO2 and respiratory rate.

We believe many more patients could benefit from such kind of monitoring in the waiting area. As they were not considered eligible for consenting to participate in the study, we did not enroll patients under the influence of alcohol or drugs or patients who suffered from disorientation for any other reason. If they can be awakened easily and have stable vital signs at the first check, these patients are usually triaged to be further observed in the waiting area. However, especially they are at risk of suffering further decline of consciousness during their stay. As they also often suffer from nausea and vomiting, we believe that mainly in these patients additional monitoring might be very valuable. Checking of SpO2 and respiratory rate can help track of their breathing at all times, making sure they are not choking and are breathing sufficiently.

One of the major limitations of our study is it's observational character, even if we tried to avoid any kind of bias be adhering strictly to our predefined inclusion and exclusion criteria and limiting the monitoring to a strictly predefined Emergency Severity Index patient group. Still there is a tiny open door left for users and in some kind also for patients in influencing the experiences reported hereby, especially considering the huge amount of patient visits per year in our department versus the few patients studied. Still we think that we could nicely show that this kind of surveillance in the waiting area of an emergency department is feasible and well accepted by patients and users. Due to the limitation of not having conducted a comparative study we also were only able report about the “duration of monitoring in minutes,” which reflects the “waiting time for admission/discharge.”

IntelliVue Guardian Solution (IGS) was originally designed for general wards and we believe it could be optimized for even better use in an emergency department. For example, continuous ECG is a very important function for a monitoring device in the waiting area to have.

## CONCLUSIONS

Continuous monitoring of potentially acutely sick patients admitted to an emergency department was feasible, easy to use, clinically effective, and could be performed with a wireless monitoring solution such as IntelliVue Guardian Solution (IGS) including cableless measurement devices.
